# 13-Valent pneumococcal conjugate vaccines vaccination innovative strategy in Weifang City, China: a case study

**DOI:** 10.1186/s40249-023-01165-1

**Published:** 2023-12-01

**Authors:** Jiachen Wang, Yujue Wang, Ruoyu Xu, Ting Zhang, Yanyan Jiang, Yuanyuan Wang, Yi Wang, Yuanze Du, Wenxue Sun, Kai Deng, Weizhong Yang, Zengwu Wang, Luzhao Feng, Chunping Wang

**Affiliations:** 1 School of Public Health, Weifang Medical University, Weifang, 261053 Shandong People’s Republic of China; 2 School of Dentistry, University of California Los Angeles, Los Angeles, CA USA; 3 School of Population Medicine and Public Health, Chinese Academy of Medical Sciences & Peking Union Medical College, Beijing, 100730 People’s Republic of China; 4 State Key Laboratory of Respiratory Health and Multimorbidity, Beijing, People’s Republic of China; 5 Key Laboratory of Pathogen Infection Prevention and Control (Peking Union Medical College), Ministry of Education, Beijing, People’s Republic of China; 6 Division of Infectious Diseases, Center for Disease Control and Prevention, Weifang, 261072 Shandong People’s Republic of China; 7 Division of Immunization, Center for Disease Control and Prevention, Weifang, 261072 Shandong People’s Republic of China; 8 Department of Public Health, Weifang People’s Hospital, Weifang, People’s Republic of China; 9 Department of Nursing, Hospital of Chengdu Office of the People’s Government of Tibet Autonomous Region, Chengdu, 610041 Sichuan Province People’s Republic of China; 10 Department of Neurosurgery, Weifang People’s Hospital, Weifang, People’s Republic of China

**Keywords:** Pneumococcal conjugate vaccine, Pneumococcal disease, Non-immunization program, Vaccination, Vaccine-preventable disease, Vaccination strategy, China

## Abstract

The World Health Organization (WHO) prioritizes pneumococcal disease as a vaccine-preventable disease and recommends the inclusion of pneumococcal conjugate vaccines (PCV) in national immunization programs worldwide. However, PCV is not included in the National Immunization Program in China and has low vaccination coverage due to its high cost. To address this, Weifang City implemented an innovative strategy for a 13-valent PCV (PCV13) on June 1, 2021. This strategy aimed to provide one dose of PCV13 free of charge for children aged 6 months to 2 years in registered households and to adopt a commercial insurance model with one dose of PCV13 free of charge in 2023 for children over 2 years old. The Health Commission of Weifang and other departments conducted a comprehensive investigation and considered various factors, such as vaccine effectiveness, safety, accessibility, vaccine price, and immunization schedules, for eligible children (under 5 years old). Stakeholder opinions were also solicited before implementing the policy. The Commission negotiated with various vaccine manufacturers to maximize its negotiating power and reduce vaccine prices. The implementation plan was introduced under the Healthy Weifang Strategy. Following the implementation of this strategy, the full course of vaccination coverage increased significantly from 0.67 to 6.59%. However, vaccination coverage is still lower than that in developed countries. Weifang's PCV13 vaccination innovative strategy is the first of its kind in Chinese mainland and is an active pilot of non-immunization program vaccination strategies. To further promote PCV13 vaccination, Weifang City should continue to implement this strategy and explore appropriate financing channels. Regions with higher levels of economic development can innovate the implementation of vaccine programs, broaden financing channels, improve accessibility to vaccination services, and advocate for more localities to incorporate PCV13 into locally expanded immunization programs or people-benefiting projects. A monitoring and evaluation system should also be established to evaluate implementation effects.

**Graphical Abstract:**

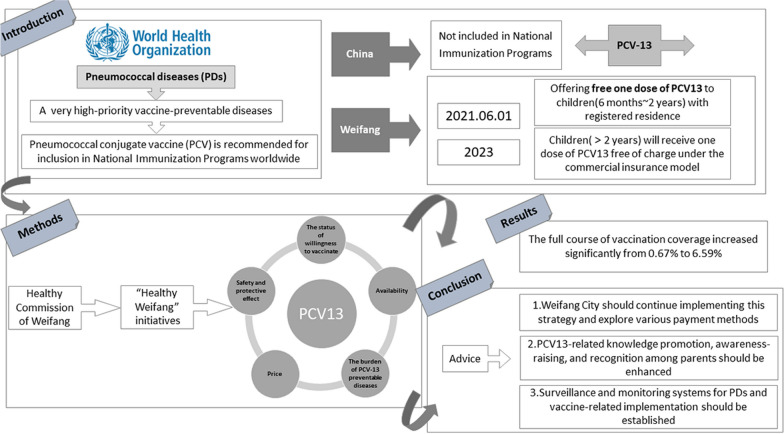

**Supplementary Information:**

The online version contains supplementary material available at 10.1186/s40249-023-01165-1.

## Background

Pneumococcal diseases (PDs), caused by *Streptococcus pneumoniae* (Spn), include pneumonia, meningitis, and acute otitis media [1]. PDs have become a serious public health problem worldwide and pose a significant economic burden on families and society [2, 3]. The use of pneumococcal conjugate vaccines (PCV) is the most cost-effective way to prevent Spn infection [4]. The World Health Organization (WHO) has listed PDs as very high-priority vaccine-preventable diseases and recommends that all countries include PCV in their National Immunization Programs (NIP) [5–7]. As of April 2021, 164 countries have included, or are planning to include, PCV in their NIPs. 13-valent pneumococcal conjugate vaccine (PCV13) is the most widely available polysaccharide conjugate vaccine for children under two years of age [8]. In China, PCV13 is not included in the NIP. Parents can choose to vaccinate their children at their own expense. However, PCV13 coverage in China is lower than the global average [9]. It is recommended that "Healthy China" initiatives include expanding the number of vaccines in the NIP to promote health equity [10]. Effective and efficient vaccination strategies are crucial for ensuring quality and quantity of vaccination services and are an important guarantee for the fair and effective implementation of the NIP. To this end, Weifang City in Shandong Province implemented an innovative strategy by offering one dose of domestic PCV13 free of charge to children aged 6 months to 2 years with registered permanent residences, which was the first pilot PCV13 vaccination program in Chinese mainland (4 doses of PCV13 are recommended for full protection when starting at 3 months of age or younger, but only 1 dose is recommended when starting at 2 years of age or older).

## Appropriate policy settings for the implementation of immunization strategies

Currently, the cost of a single domestic dose of PCV13 in Weifang City is 630 Chinese Yuan (CNY). This is too high for many parents, and the points of vaccination in community health centers and township hospitals have been reported to the health authorities and the Centers for Disease Control and Prevention (CDC) in Weifang City. To investigate the willingness of parents to vaccinate their children with PCV13 or pay for PCV13, a survey was conducted in Weifang City. This survey showed that 76.87% of parents were willing to pay all the cost [11]. Considering the survey findings, the Health Commission of Weifang organized an investigation of public opinion by experts. Furthermore, the Health Commission of Weifang and other departments formulated the "Healthy Weifang Health Knowledge Popularization Action (2020–2022)", which includes a plan to actively vaccinate high-risk groups against influenza and PDs and advocate for the inclusion of influenza and pneumococcal vaccines in local public health service projects. In recent years, the People’s Government of Weifang City implemented several free vaccination strategies for non-NIP vaccines, such as the free chickenpox vaccination program for children and free influenza vaccination program for persons over 70 years of age. Based on this experience, the People’s Government of Weifang City plans to launch a PCV13 innovative vaccination strategy by 2021. From June 1, 2021, children aged 6 months to 2 years with registered permanent residence in Weifang City could receive a free dose of PCV13 at the vaccination sites in their jurisdiction, and children with non-local household registration who have been continuously managed by the vaccination information system of Weifang City for over 3 months can also receive a free dose of PCV13. Finally, from March 21, 2023, the People’s Government of Weifang City plans to implement the Weifang Social Welfare Insurance, which reimburses the families of children over two years old for one dose of PCV13.

## Factors to consider when developing a PCV13 vaccination strategy

### Effectiveness and safety of the vaccines

Vaccination is an effective measure to prevent Spn infections and reduce the formation of drug-resistant strains [12]. A study conducted in Ningbo City, China evaluated the effectiveness of PCV13 in children with pneumonia under two years of age. The effectiveness was 57.80% in children who received four doses of PCV13 and 25.00% in children who received three doses [8]. In the United States, PCV13 had an effectiveness of 60.20% against invasive PD (IPD) of any etiology [13]. The introduction of PCV13 in the United States resulted in a significant decrease in IPD, including non-antibiotic-susceptible IPD, in multiple age groups [14, 15]. Safety is a top priority in immunization programs, and only safe vaccines can be used in expanded programs on immunization (EPI). Furthermore, studies have shown that PCV13 has better immunogenicity, efficacy, and safety than PCV7 and PCV9 [16]. Owing to the effectiveness and safety of PCV13, the People’s Government of Weifang City and the Health Committee of Weifang decided to provide one free dose of PCV13 to eligible children.

### The burden of PCV-13 preventable diseases

The number of pneumococcal disease cases in children under 5 years old in China accounts for 12.00% of the world’s total number of cases, thus making China the second country in the world with the highest occurrence rate. The WHO estimated that in 2015 there were about 210,000 severe cases and up to 7000 deaths among PDs in children under 5 years of age in China [12]. According to the China Health Statistical Yearbook 2018, 3.59 million people were discharged from hospitals with pneumonia in 2017, with a case fatality rate of 0.49%, and 60.70% of the discharged medical records were children < 5 years old [17]. In the 290 cases of children with acute otitis media in Xi 'an in 2014, the detection rate of Spn was 18.73% [18]. Based on the above data, the Weifang Municipal government believes that the burden of pneumococcal diseases in children is more serious, and limited health resources should be used to reduce pneumococcal diseases.

### Vaccine availability

PCV13 production technology is relatively mature in China. Imported PCV13 was first introduced in China in March 2017, primarily targeting infants and young children aged between 6 weeks to 15 months. A domestic PCV13 supply was launched in 2020 and is used for infants aged between six weeks and five years. The production capacity of domestic vaccines is adequate and the price of domestic vaccines is lower than that of imported vaccines. According to the provisions of the Government Procurement Law, the People’s Government of Weifang City procured domestic PCV13 for free immunization programs. Imported PCV13 is not eligible for use under the free policy, although the administration of imported vaccines is continued for children who have already received imported vaccines to ensure that the same vaccine is used. Vaccine manufacturers prepare for vaccine production in advance based on the total quantity purchased by the government to ensure a sufficient supply of PCV13.

### The status of willingness to vaccinate

With the awareness of disease prevention increasing, the need for vaccination is gradually being recognized. A meta-analysis of the vaccination willingness of pneumococcal vaccines in Chinese residents showed that 62.00% of caregivers expressed willingness to have their children receive pneumococcal vaccines [19]. The willingness of parents to vaccinate family members with pneumococcal vaccines in Anyang city found that 83.90% of parents were willing to vaccinate family members with pneumococcal vaccines [20]. The preceding investigation, conducted by our research team, which scrutinized the proclivity of caregivers to vaccinate children with pneumococcal vaccines and their inclination to bear associated costs within a resource-constrained milieu in China, yielded findings indicating that within the study cohort, comprising 899 individuals (71.69%) who exhibited vaccine acceptance, 254 individuals (20.26%) characterized by vaccine hesitancy, and 101 individuals (8.05%) displaying vaccine refusal, there exists a substantial local demand for the administration of PCV13 vaccination among children under the age of five [21].

### Price of vaccines and the government's financial capacity

The People’s Government of Weifang City has adopted a drug bidding and procurement model as a basis for establishing a mechanism that combines the payer and purchaser roles, allowing for stronger negotiation power to achieve affordable pricing for PCV13. This approach resulted in a price reduction from the normal price of CNY 730 to CNY 630 through negotiation between the government and vaccine manufacturers. The People’s Government of Weifang City estimated the birth of 62,000 children in 2021 and based on one dose of free PCV13 for each child, vaccine costs were calculated at 39.06 million CNY, along with a vaccination service fee of 1.36 million CNY, totaling 40.42 million CNY. The total special expenditure budget of the People’s Government of Weifang City for PCV13 was 40 million CNY in 2021. To meet the demand for vaccines, county-level CDCs in Weifang City purchased 167,502 domestic PCV13 in 2021, based on estimations.

## Organization and implementation of vaccination

County-level CDCs play a critical role in ensuring that vaccination programs are delivered effectively and safely. City- and county-level CDCs organize vaccination units to provide professional training on vaccination specifications, procedures, and techniques to minimize the risk of vaccination accidents and mistakes. To maintain the potency of vaccines, it should be ensured that vaccines are stored and transported at specific temperatures. The cold chain system is an essential part of vaccine management, and the effective management of cold chain equipment is crucial to ensure that vaccines remain viable and effective. To this end, vaccination units rely on refrigerators, refrigerated bags, and other equipment to ensure cold-chain storage, transportation, and use of vaccines. In Weifang City, all vaccination clinics provide a free vaccination with a single dose of PCV13 to eligible children. In addition to providing free vaccination, public health authorities use mainstream and new media channels to promote the significance of PCV13 vaccination and the government's policy on free vaccination, with the aim of increasing public awareness and encouraging more people to take advantage of the available vaccination services. One free dose of vaccinations and multi-purpose advocacy has contributed significantly to the success of this strategy.

## Implementation status

In the pre-implementation phase of the PCV13 innovative vaccination strategy, the PCV13 vaccination coverage for children born in Weifang City between June 1, 2016, and May 31, 2021, was low, with only 3973 children completing the full four immunization schedules, corresponding to a vaccination coverage of 0.67%. However, after the introduction of this innovative strategy, the number of children completing the full course of PCV13 increased significantly to 39,112, with a corresponding coverage of 6.59%. Prior implementation of the strategy, 12 of the 15 counties and urban areas in Weifang City had fewer than 10 children aged 2 to 6 months vaccinated with PCV13. In contrast, following the introduction of the innovative strategy, 5 of the 15 counties and districts in Weifang City had over 100 children aged 2 to 6 months vaccinated with PCV13. Among the 593,784 children included in the study, 77,072 (12.98%) received the free PCV13 vaccination (Table 1).

**Table 1:** Domestic 13-valent pneumococcal conjugate vaccines use under Weifang childhood immunization programs before and after the innovative immunization strategy

Immunization procedures	1 dose, *n*	2 doses, *n*	3 doses, *n*	4 doses, *n*	Full vaccination rate, *n* (%)
Before the strategy					3973 (0.67)
2–6 months old	86	123	358	130	
7–11 months old	90	175	153		
12–23 months old	826	1208			
2–5 years old	2482				
Total	3484	1506	511	130	
After the strategy					39,112 (6.59)
2–6 months old	18	50	58	561	
7–11 months old	488	133	438		
12–23 months old	37,213	14,737			
2–5 years old	23,376				
Total	61,095	14,920	496	561	

4 doses of vaccine were considered to be a full immunization for children aged 2–6 months

## Challenges in the implementation of PCV13 vaccination innovative strategies

We conducted interviews with personnel from the Centers for Disease Control and Prevention (CDCs) and healthcare practitioners at vaccination clinics situated in Weifang City. Several challenges were identified during the implementation of this PCV13 vaccination innovative strategy. First, children had a high likelihood of experiencing the "dropped needles" phenomenon, whereby children received only one free dose of the vaccine, and subsequently failed to complete the full course of immunization due to financial constraints. This leads to low vaccination rates and poor immune responses. Second, parents in rural areas have limited access to educational resources, resulting in poor health awareness. In addition, inadequate promotion of the vaccine by vaccination units made it difficult to conduct vaccination campaigns in rural areas, resulting in low coverage. Third, the staff of the vaccination units was overburdened due to limited human resources, which impeded the delivery of vaccination knowledge to parents and hindered the effective promotion of the PCV13 vaccination innovative strategy. For example, some parents believe that vaccination will cause certain side effects. Medical institutions therefore need to strengthen the provision of accurate information about vaccines to guide public opinion. Vaccination sites should optimize the management of childhood vaccination schedules to avoid missing doses. Further challenges resulted from the coronavirus disease 2019 (COVID-19) pandemic as human and financial resources used to implement the innovative PCV13 vaccination strategy were transferred to support the response to the COVID-19 pandemic. In addition, the COVID-19 pandemic and stay-at-home orders led to an unprecedented decrease in the administration of routine vaccines.

## Discussion

PCV vaccination is an effective and cost-efficient measure for preventing PDs [22]. The execution of the innovative PCV13 vaccination strategy by the People's Government of Weifang City has proven initially efficacious. Subsequent to the strategy's implementation, a noteworthy escalation in vaccination coverage among children was evident across all counties within Weifang City. Specifically, full-course vaccination coverage ascended significantly from a mere 0.67% to a commendable 6.59%. Furthermore, it is worth noting that only 29.70% of the participants exhibited hesitancy towards PCV13 vaccination for their children, a figure that is notably lower when juxtaposed with hesitancy rates documented in prior research endeavors concerning varicella vaccine (51.20%), enterovirus 71 inactivated vaccine (33.84%), and HPV vaccine (37.20%) [2]. Additionally, 70.51% of caregivers expressed willingness to have their children receive pneumococcal vaccines under the aegis of the one-free-dose policy in Weifang. The acceptance rate observed in our study surpasses analogous field surveys conducted in Shanghai and Guangzhou [23]. The implementation of a PCV13 vaccination innovative strategy by the People’s Government of Weifang City, including offering one dose of PCV13 free of charge and exploring commercial insurance models, can serve as a valuable experience for other regions planning to provide free PCV13. This case study presents the details of the implementation process and initial outcomes of the PCV13 vaccination strategy in Weifang City. Several valuable insights can be gained from this study for policymakers and public health professionals considering the implementation of similar vaccination strategies in other regions.

First, we found that the vaccination coverage of children aged 5 years significantly increased after the implementation of the strategy, although it was still comparatively lower than that in developed countries. This study provides only preliminary insights into the effectiveness of innovation strategies in the early stages. Some parents are hesitant to vaccinate their children because of financial constraints as PCV13 is not included in China’s NIP. The procurement price of PCV13 in China is much higher than that of international organizations, such as United Nations International Children's Emergency Fund (UNICEF). While UNICEF’s procurement price is only USD 3.30, the price range in China is between USD 67.59–101.17 [24]. Each dose of the domestic vaccine costs approximately CNY 630, equivalent to approximately USD 91, based on the current exchange rate. The cost of administering a single dose of PCV13 or completing the entire vaccination schedule is comparatively high. A comparative analysis of the multi-agent co-payment financing mechanism of the four doses of children's PCV13 in Weifang City shows that when the purchase price drops to CNY 456.40, the individual out-of-pocket cost is zero. Therefore, we propose that the People’s Government of Weifang City focus on financing mechanisms that reduce vaccine prices, and which involves three approaches: (1) The city should explore reasonable and feasible financing methods such as government sharing mechanisms, commercial insurance, society, and private individuals. This may include the incorporation of PCV13 into medical and commercial insurance systems. (2) Centralized procurement, which integrates the payer and purchaser to maximize negotiations and make the price of PCV13 affordable, should be adopted to reduce the cost of vaccine procurement. (3) Manufactures should be encouraged to develop novel vaccines and technologies at lower costs to increase PCV13 coverage.

The second key lesson identified was that health authorities should strengthen vaccine publicity for parents of children through multiple channels, popularize knowledge of PD prevention and treatment, and increase awareness and acceptance of PCV13. Medical personnel are always listed as the most trustworthy sources of vaccine-related recommendation information [25]. In a contemporaneous article published in *China CDC Weekly* in 2022, parental inclination to vaccinate their children with PCV13 or incur expenses related to PCV13 vaccination was appraised. This inquiry also delved into the pivotal role played by healthcare professionals in amplifying vaccine acceptance and augmenting the willingness of the general populace to partake in vaccination. The study revealed that 94.38% of participants conveyed trust in vaccine information disseminated by healthcare practitioners, and this cohort exhibited significantly greater willingness in contrast to those who harbored reservations regarding information imparted by healthcare professionals [23]. Therefore, the publicity approach should include medical staff conducting standardized training, encouraging medical staff to learn communication skills, and providing information on the safety and effectiveness of PCV13 in vaccinated groups. During the campaign, the Health Commission of Weifang and the CDC released information on free PCV13 vaccination on their official websites and WeChat public accounts, popularized PCV13 and medical knowledge of PDs, and provided information on precautions and contraindications for vaccination. Vaccination units informed parents of eligible children in their jurisdictions about relevant policies and routine immunization schedules via telephone. It is worth highlighting that this intervention strategy has proven to be efficacious within the context of Weifang's experience.

The third key lesson was the importance of standardizing the vaccination procedure and providing guidance to ensure effective implementation. All stakeholders in China strictly follow regulations and laws [26]. Manufacturers should be strict in quality control of production and supply. City- and county-level CDCs purchased PCV13 from statutory channels, and standardized training on specific implementation plans was conducted for all vaccination sites. These measures ensure smooth implementation of the vaccination service and contribute to the overall success of the PCV13 vaccination innovative strategy.

Additionally, countries or areas that are introducing PCV for the first time should establish surveillance systems for PDs burden and monitor the impact of vaccination programs [27]. Therefore, the People’s Government of Weifang City should reinforce the surveillance and monitoring of PDs in the future.

The implementation of the PCV13 innovative immunization strategy in Weifang is a good pilot and initial strategy in Chinese mainland, which represents an important exploration of non-NIP vaccines. This long-term strategy can significantly reduce the morbidity, mortality, hospitalization duration, and medical costs, thereby providing a cost-effective solution for preventing PCV-related diseases.

In addition, the experiences learned from this vaccination demonstration project, particularly in communication campaign logistics arrangements and vaccine administration are consistent with the experience of many countries that have included PCV13 in their immunization programs, such as India and Indonesia. However, Weifang has made a new exploration in multi-agent copayment financing mechanisms of vaccines. This is different from countries that have included PCV13 in their immunization programs, with the help of the Global Alliance for Vaccines and Immunization. Different regions may have unique experiences and models for implementing novel vaccine strategies; however, each region should consider its own circumstances, learn from the pilot project’s implementation experiences, and develop a feasible plan for the local context.

## Conclusions

The implementation of the PCV13 innovative vaccination strategy by the People’s Government of Weifang City has been effective initially. Weifang City should continue implementing this strategy and explore various payment methods, such as installment payments, inclusion of PCV13 in medical insurance, and financial subsidies. The free dose of PCV13 should be appropriately increased to obtain better protective effects. Simultaneously, surveillance and monitoring systems for PDs and vaccine-related implementation should be established to evaluate their effectiveness and impact. Moreover, PCV13-related knowledge promotion, awareness-raising, and recognition among parents should be enhanced. Finally, this strategy provides an experience in adopting innovative implementation paths, broadening financing channels, improving the convenience of vaccination services, incorporating PCV vaccination into local EPI projects, and gradually increasing vaccination rates. As the strategy has been implemented for a relatively short period of time, long-term success of the program remains unclear, it may be a very good progress, but may not be a great success (Additional file 1).

### Supplementary Information

**Additional file 1: Table S1.** Basic situation of PCV13 vaccination for children aged under 5 years in Weifang before and after the innovative immunization strategy. **Table S2.** Full vaccination rate of children with domestic PCV13 by district/county before and after the innovative immunization strategy.

## Data Availability

The datasets generated and analyzed in the current study are not publicly available because of the regulations of the Weifang CDC. Readers of the article should approach the Weifang CDC and obtain permission for the release of the datasets.

## Abbreviations

PDs: Pneumococcal disease
SPn: *Streptococcus pneumoniae*
PCV: Pneumococcal conjugate vaccine
WHO: World Health Organization
NIP: National Immunization Program
PCV13: 13-Valent pneumococcal conjugate vaccine
CNY: Chinese Yuan
CDC: Center of Disease Control and Prevention
IPD: Invasive Pneumococcal Disease
EPI: Expanded Program on Immunization
COVID-19: Coronavirus disease 2019
UNICEF: United Nations International Children's Emergency Fund
USD: United States dollar
