# Supplementary material for: 13-Valent pneumococcal conjugate vaccines vaccination innovative strategy in Weifang City, China: a case study
Source: Infect Dis Poverty. 2023 Dec 1;12:110. doi: 10.1186/s40249-023-01165-1 (PMC10691032; doi:10.1186/s40249-023-01165-1)
Supplement: Supplementary file 1 — Additional file 1: Table S1. Basic situation of PCV13 vaccination for children aged under 5 years in Weifang before and after the innovative immunization strategy. Table S2. Full vaccination rate of children with domestic PCV13 by district/county before and after the innovative immunization strategy. [file 40249_2023_1165_MOESM1_ESM.docx]

ADDITIONAL MATERIAL

These materials provided details for achievement by this strategic.

Here are the results from our upcoming independent article in print.

**1. *Chunping Wang.* Impact of innovative immunization strategy on PCV13 vaccination coverage among children under 5 years in Weifang city, China: a retrospective study. (in the process of submitting a manuscript)**

Among the 593,784 children included in the study, the PCV13 vaccination rate in Weifang was generally low before the innovative immunization strategy. However, after the introduction of this innovative strategy, each dose of vaccine has changed significantly. The 1 dose of vaccination coverage increased significantly from 075% to 10.29%. Rural 1-dose PCV13 vaccination rate was higher than urban; After the vaccination program, the PCV13 coverage of children increased significantly in all counties within Weifang City (*P*<0.001). Among them, Gaoxin District, Gaomi City and Binhai Economic and Technological Development Zone had the largest increase in the full vaccination rate（**Table S1**&**Table S2**）.

**Table S1** **Basic situation of PCV13 vaccination for children aged under 5 years in Weifang before and after the innovative immunization strategy**

| Variables | Number | 1 dose (n, %) | 2doses (n, %) | 3 doses (n, %) | 4 doses (n, %) | Χ^2^ | *P*-value |
| --- | --- | --- | --- | --- | --- | --- | --- |
| Before the strategy | | | | | | | |
| Total population | 593784 | 4450 (0.75) | 2242 (0.38) | 3438 (0.58) | 10218 (1.72) |  | |
| Sex |  |  |  |  |  |  | |
| Male | 310284 | 2333 (0.75) | 1211 (0.39) | 1755 (0.57) | 5294 (1.71) | 5.34 | 0.149 |
| Female | 283500 | 2117 (0.75) | 1031 (0.36) | 1683 (0.59) | 4924 (1.74) |  |  |
| Census register types | | | | | | | |
| Urban | 458772 | 3949 (0.86) | 2004 (0.44) | 3332 (0.73) | 10108 (2.20) | 923.32 | <0.001 |
| Rural | 135012 | 501 (0.37) | 238 (0.18) | 106 (0.08) | 110 (0.08) |  |  |
| Date of Birth |  |  |  |  |  |  |  |
| 2016.6.1-2017.5.31 | 178578 | 215 (0.12) | 52 (0.03) | 123 (0.07) | 181 (0.10) | 4134.87 | <0.001 |
| 2017.6.1-2018.5.31 | 138755 | 824 (0.60) | 167 (0.12) | 405 (0.29) | 1287 (0.93) |  |  |
| 2018.6.1-2019.5.31 | 109266 | 2067 (1.89) | 562 (0.51) | 567 (0.52) | 3489 (3.19) |  |  |
| 2019.6.1-2020.5.31 | 97202 | 1199 (1.23) | 1281 (1.32) | 1493 (1.54) | 5261 (5.41) |  |  |
| 2020.6.1-2021.5.31 | 69983 | 145 (0.21) | 180 (0.26) | 850 (1.21) | 0 (0.00) |  |  |
| After the strategy |  |  |  |  |  |  |  |
| Total population | 593784 | 61098 (10.29) | 14925 (2.51) | 540 (0.09) | 1537 (0.26) |  |  |
| Sex |  |  |  |  |  |  |  |
| Male | 310284 | 31984 (10.31) | 7848 (2.53) | 299 (0.10) | 765 (0.25) | 6.39 | 0.094 |
| Female | 283500 | 29114 (10.27) | 7077 (2.50) | 241 (0.09) | 772 (0.27) |  |  |
| Census register types |  |  |  |  |  |  |  |
| Urban | 458772 | 46754 (10.19) | 12996 (2.83) | 470 (0.10) | 1480 (0.32) | 1110.72 | ＜0.001 |
| Rural | 135012 | 14344 (10.62) | 1929 (1.43) | 70 (0.05) | 57 (0.04) |  |  |

**Table S2** Full vaccination rate of children with domestic PCV13 by district/county before and after the innovative immunization strategy

| District/County | GDP (billion CNY) | Before the strategy | After the strategy |
| --- | --- | --- | --- |
| Kuiwen | 361.7 | 317(1.12) | 1959(6.92) |
| Weicheng | 364 | 115 (0.30) | 2299 (5.95) |
| Hanting | 270.3 | 136 (0.41) | 1778 (5.35) |
| Fangzi | 211.6 | 318 (0.86) | 1665 (4.51) |
| Qingzhou | 676.8 | 174 (0.30) | 3580 (6.11) |
| Zhucheng | 767.4 | 198 (0.32) | 3201 (5.14) |
| Shouguang | 953.6 | 809 (1.06) | 5386 (7.04) |
| Anqiu | 403.9 | 315 (0.63) | 2307 (4.64) |
| Gaomi | 614.6 | 479 (0.97) | 5085 (10.25) |
| Changyi | 527.9 | 231 (0.97) | 1059 (4.46) |
| Linqu | 389.4 | 75 (0.16) | 2270 (4.70) |
| Changle | 405.3 | 553 (1.43) | 1962 (5.09) |
| Gaoxin | 615.7 | 148 (0.42) | 5652 (16.0) |
| Binhai | 336.9 | 99 (1.19) | 674 (8.12) |
| Xiashan | 50.3 | 6 (0.10) | 235 (4.06) |

**2. *Chunping Wang*. Comparative analysis on the multi-agent co-payment financing mechanism of four doses of children's PCV13 in Weifang city, China.(in the process of submitting a manuscript)**

In this study ,we followed previous domestic and foreign experience indicating a decrease in the price with mass purchase, and thus considers different prices of PCV13 to analyze the herd immunity effect. The purchase price is assumed to decrease from the current CNY652 in Weifang, with CNY100 as the decline range, and incorporates the purchase price of a domestic manufacture and Pan American Health Organization (PAHO) to compute the total cost of achieving herd immunity and the cost borne by each financing channel under different scenarios.

The vaccination rate is observed to increase as the out-of-pocket payments for individuals decrease. In the case of the "individual - commercial insurance - central and local finance co-payment" approach, children who receive four doses of PCV13 vaccination are required to bear 30% of the cost, leading to an estimated coverage rate of 47.4%. However, in the "commercial insurance - central and local finance co-payment" and "commercial insurance and local finance co-payment" approaches, the burden of out-of-pocket payments is removed, resulting in an expected coverage rate of 72.3% for four doses of PCV13 vaccination.

1. ***Chunping Wang*. Willingness to pay for 13-valent pneumococcal conjugate vaccine and factors influencing willingness among parents of children under 3 months of age in Weifang city.**

https://doi.org/10.19914/j.CJVI.2023102

1. ***Chunping Wang*. Willingness of Caregivers to Have Their Children Vaccinated with Pneumococcal Vaccines in the Context of an Innovative Immunization Strategy - Weifang City, Shandong Province, 2021. China CDC Wkly. 2022; 4(20): 421-425.**

https://doi.org/10.46234/ccdcw2022.097.

1. **Chunping Wang. Factors associated with PCV13 vaccine hesitancy in parents under an innovative immunization strategy: a cross-sectional study. Weifang City: Shandong Province.China;2021. China CDC Wkly. 271–277.**

<https://doi.org/10.46234/ccdcw2023.049>;5; 2023

**6. *Luzhao Feng*. Caregiver Willingness to Vaccinate Children with Pneumococcal Vaccines and to Pay in a Low-Resource Setting in China: A Cross-Sectional Study. Vaccines, 10(11), 189**

<https://doi.org/10.3390/vaccines10111897>

**7. *Chunping Wang*. Economic evaluations of 13-valent Pneumococcal Conjugate Vaccine: a systematic review. Expert Rev Vaccines. 2023;22:193–206.**

<https://doi.org/10.1080/14760584.2023.2173176>
